# Unilateral Hindlimb Casting Induced a Delayed Generalized Muscle Atrophy during Rehabilitation that Is Prevented by a Whey or a High Protein Diet but Not a Free Leucine-Enriched Diet

**DOI:** 10.1371/journal.pone.0070130

**Published:** 2013-08-27

**Authors:** Hugues Magne, Isabelle Savary-Auzeloux, Carole Migné, Marie-Agnès Peyron, Lydie Combaret, Didier Rémond, Dominique Dardevet

**Affiliations:** 1 Clermont Université, Université d'Auvergne, Unité de Nutrition Humaine, BP 10448, F-63000 Clermont-Ferrand, France; 2 INRA, UMR 1019, UNH, CRNH Auvergne, F-63000 Clermont-Ferrand, France; Paris Institute of Technology for Life, Food and Environmental Sciences, France

## Abstract

Sarcopenia is the general muscle mass and strength loss associated with ageing. Muscle atrophy could be made worse by exposure to acute periods of immobilization, because muscle disuse by itself is a stimulus for atrophy. Using a model of unilateral hindlimb casting in old adult rats, we have already demonstrated that the primary effect of immobilization was atrophy in the casted leg, but was also surprisingly associated with a retarded atrophy in the non-casted leg during rehabilitation. In search of mechanisms involved in this generalized atrophy, we demonstrated in the present study that contrary to pair-fed non-immobilized control animals, muscle protein synthesis in the non-immobilized limb was unable to adapt and to respond positively to food intake. Because pair-fed control rats did not lose muscle mass, this defect in muscle protein synthesis may represent one of the explanation for the muscle mass loss observed in the non-immobilized rats. Nevertheless, in order to stimulate protein turn over and generate a positive nitrogen balance required to maintain the whole muscle mass in immobilized rats, we tested a dietary free leucine supplementation (an amino acid known for its stimulatory effect on protein metabolism) during the rehabilitation period. Leucine supplementation was able to overcome the anabolic resistance in the non-immobilized limb. A greater muscle protein synthesis up-regulation associated with a stimulation of the mTOR signalling pathway was indeed recorded but it remained inefficient to prevent the loss of muscle in the non-immobilized limb. By contrast, we demonstrated here that whey protein or high protein diets were able to prevent the muscle mass loss of the non-immobilized limb by sustaining muscle protein synthesis during the entire rehabilitation period.

## Introduction

Sarcopenia is an age-related loss in skeletal muscle mass and strength associated with normal ageing [1]. Besides a slow and progressive loss over years, English & Paddon-Jones have suggested that, when ageing, sarcopenia could also result from muscle atrophy episodes followed by uncompleted muscle recovery [2]. The authors named this phenomenon the ‘catabolic crisis model’ which has been observed previously after generalized catabolic states including food deprivation [3] or glucocorticoids treatment [4]. We have also recently shown that contrarily to adults [5], muscle mass loss was not recovered after 40 days of rehabilitation in an old adult rat model of unilateral hindlimb casting. More surprisingly, we showed for the first time that atrophy also occurred in the non-immobilized leg during the rehabilitation period and reached a non-negligible level of 10–15% decrease after the cast removal [5]. Considering that this general atrophy occurred later during the rehabilitation period (i.e. almost three-time the duration of the immobilization period after the removal of the casts), it seems unlikely that a reduction in physical activity related to immobilization may be responsible for the general atrophy observed. Furthermore, this phenomenon has not been demonstrated in young adult animals by using the same model of muscle immobilization [6] and seems then specific to ageing. Therefore, if the primary effect of unilateral immobilization results in local atrophy of the casted limb in the old adults, it could have subsequent consequences by inducing a general muscle mass loss as observed in generalized catabolic states. The consequences of this observed phenomenon may be similar to the frailty syndrome described as a physiological state of susceptibility that places older individuals at high risk for adverse out-comes such as falls, disability, morbidity and institutionalization [7]–[10].

Presently, the mechanisms involved in this delayed atrophy are nevertheless unknown. It is however noteworthy to mention that Chen et al. have shown that casting in adult volunteers affected not only the immobilized limb but also the gene expression in the non-immobilized limb. The genes affected were involved in stress response, sarcomere structure, cell growth/death and interestingly protein turnover regulation [11]. The size of the muscle compartment is indeed dependent on the overall balance between muscle protein synthesis and protein breakdown. However, we have shown that muscle proteolysis and apoptotic processes remained unaffected in the non-immobilized leg during the immobilization by casting and the following recovery period [5]. The delayed muscle mass loss observed in this non-immobilized leg seems to be linked to a depressed protein synthesis. However, this has not been demonstrated yet. Muscle protein synthesis is not constant during the day and it is subjected to variations especially following dietary intake. Amino acids are particularly efficient in stimulating muscle protein synthesis and by inhibiting muscle protein breakdown [12]–[14], hence resulting in a positive post-prandial nitrogen balance required to maintain the muscle mass constant. Among amino acids, leucine is particularly known for its ‘signal’ properties i.e. it is known to acutely increase muscle protein synthesis and decrease protein breakdown under both *in vitro* and *in vivo* conditions [15]–[24]. Leucine ingestion/infusion results in the phosphorylation of proteins critical for the anabolic mTOR signalling pathway leading to the stimulation of the initiation of muscle protein synthesis [25]–[27].

In our previous study in old adult rats [28], we have shown that either leucine supplementation incorporated into a casein diet, whey protein or a high protein diets were more efficient than a standard diet at stimulating post-prandial muscle protein synthesis in a limb that was previously immobilized by casting. However, only the whey proteins or the high protein diet were efficient to improve muscle mass recovery. The objective of the present study was to understand the mechanisms involved in the atrophy observed in the non-immobilized leg after immobilization-induced atrophy, muscle protein synthesis and key intracellular signalling factors were measured in the same animals that we previously studied [28]. We also wanted to explore kinetically the response to the diets already tested on the immobilized limb in the non-immobilized leg muscle.

## Materials and Methods

### Animals and experimental design

All procedures were performed in strict accordance with the institutional guidelines on animal experimentation in France (Animal facilitiy scientific committee, CSU INRA Clermont-Theix). At the time of the experiment, ethics committee approval was not required in France but required the official authorization for animal experimentation for the experimenter (Direction Départementale des Services Vétérinaires (DDSV), Dominique Dardevet; Authorization 63-08) and the official authorization of our animal facility (Installation Expérimentale de Nutrition (Unité de Nutrition Humaine, INRA de Theix), agreement n° C 63345.14 delivered the 22 December 2009). From 2013, France is now under the European legislation in animal ethics and all protocols should be approved by the “Comité d'Ethique En Matière d'Expérimentation Animale Auvergne (CEMEAA)”. Similar protocols with casted rats (adults or old animals) have been submitted and received the approval (CE4-09 ; CE29-11; CE108-12). All casting were performed under gas anesthesia (Fluothane). Animals were weighted every day, food intake monitored and casts were checked twice a day (morning and afternoon). If necessary, the cast was removed under gas anesthesia and replaced to insure no injury. Every effort was made to minimize suffering and animals were removed from the experiment if their food consumption was abnormal for more than 3 days.

#### Experiment 1

Male Wistar rats aged 22–24 months were housed individually under controlled environmental conditions (room temperature 22°C; 12 h light-dark cycle, light period starting at 08:00 h), fed *ad libitum* a standard 13% casein diet (**Table 1**) and given free access to water.

**Table 1:** Composition of standard and experimental diets.

	Standard diet	Control and experimental diets			
		CONTROL	LEU	WHEY	HIGH PROT
**Ingredients**					
Casein	166	166	166	-	156
Whey	-	-	-	144	144
L-cystine	1.8	1.8	1.8	-	-
Alanine^1^	-	59		-	-
Leucine	-	-	44.5	-	-
Valine^2^	-	-	5.1	-	-
Isoleucine^2^	-	-	9.8	-	-
Proline	-	-		5.7	-
Choline	-	-		2.5	2.5
Rapeseed oil	30	30	30	30	30
Peanut oil	27	27	27	27	27
Sunflower oil	3	3	3	3	3
Cellulose	35	35	35	35	35
Sucrose	100	100	100	100	100
Lactose	134	134	134	134	134
Wheat flour	458.2	399.2	398.8	482	334
Mineral mixture AIN93	35	35	35	35	35
Vitamin mixture AIN93	10	10	10	10	10
*Given during*	*adaptation/immobilization*	*recovery*	*recovery*	*recovery*	*recovery*

Quantities are expressed in g.kg^−1^ dry matter for the CONTROL, LEU, WHEY and HIGH Prot groups.

LEU, leucine; WHEY, whey protein; HIGH PROT, high protein.

1 Alanine was included in the control diet to render the diets isonitrogenous. This amino acid has no effect on muscle protein metabolism.

2 Valine and isoleucine were included in the leucine-supplemented diet to prevent the fall of their plasma concentrations induced by leucine supplementation.

After a 3-week adaptation period, seventeen rats were studied as a reference point before the immobilization period (I0) and 144 rats were anesthetized with isoflurane inhalation and subjected to unilateral hindlimb cast immobilization with an Orfit-soft plaque (Gibaud, France) for 8 days (I8) to generate local muscle atrophy. The foot joint was casted with a 130° angle and *gastrocnemius* muscle was immobilized in the shortened position. All rats were fed the standard 13% casein diet during immobilization which is known to cover the dairy protein recommendation in non-growing rats [29]. For muscle recovery, casts were removed and the rats were allowed to recover for 10 (R10), 20 (R20), 30 (R30) or 40 (R40) days. Half of the rats were fed a control diet (i.e. with casein as protein source) and constituted the CONTROL group; the other half was fed with a 4.45% leucine-supplemented diet (**Table 1**) and was the LEU group. To prevent the fall of plasma valine and isoleucine concentrations induced by leucine supplementation, the leucine-supplemented diet was also supplemented with appropriate amounts of these amino acids [30]. Alanine, an amino acid that has no effect on muscle protein metabolism, was included in the control diet to render the diets iso-nitrogenous and iso-caloric.

Before immobilization (I0) and at the end of the immobilization (I8) or recovery periods (R10, R20, R30, R40), animals were fasted overnight (light period) and euthanized the next morning under pentobarbital sodium anaesthesia (50 mg/kg ip). On the morning of each time point studied, half of the rats in each group were not fed, so that they were in a post-absorptive state. These rats were assigned the acronym “PA” (PA CONTROL and PA LEU). The others ate as usual for 1 h and then were in the post-prandial state as previously described [16], [30]; they were named “PP” (PP CONTROL and PP LEU). The food consumption was identical between all groups at R20 (8.8±0.6, 8.4±0.6, 7.4±0.6 and 7.4±0.5 g for control, leucine, whey and high protein diet groups, respectively) and R40 (7.7±0.7, 8.1±0.6, 7.5±0.5 and 8.1±0.6 g for control, leucine, whey and high protein diet groups, respectively).

Finally, for immobilized rats, the number of animals was n = 8 for each group and each nutritional state at each time point. Non-casted pair-fed rats were also studied at the PA and the PP states using the same procedure (n = 8 per group at each time point). They were fed with the standard diet from I0 to I8 and then with the control diet during the recovery.

#### Experiment 2

In a second experiment designed exactly as Experiment 1, the effect of whey protein or high protein diets was assessed on the non-immobilized limb in rats (described in [28]). Forty one animals were subjected to cast immobilization for 8 days and then allowed to recover during 20 (R20) and 40 (R40) days. Eight animals were euthanized at I8 and the others were divided in two groups: the first one (n = 16) received a whey protein diet WHEY group) and the second group (n = 17) received a high protein diet (HIGH PROT group) (**Table 1**). Both diets are iso-caloric and contain same calories as others diets. Seven animals were used as a control before casting (I0). All animals were studied at the post-prandial state.

### Measurement of proteasome activities

In this pathway, two distinct steps are depicted: 1) the ubiquitination of proteins and 2) their degradation by the 26S proteasome. We have firstly investigated the chrymotrypsin- and the trypsin-like activity of the proteasome which are surrogate markers of this pathway.

One hundred and fifty mg of *gastrocnemius* muscle powder from non-immobilized muscles at each time point were homogenized in 10 volumes of an ice-cold buffer containing 50 mm Tris-Cl (pH 7.5), 5 mm MgCl_2_, 250 mm sucrose, 1 mm Dithiothreitol (DTT), 10 nm adenosine triphosphate (ATP) and protease inhibitors (10 µg/ml antipain, 10 µg/ml leupeptin, 10 µg/ml aprotinin, 10 µg/ml pepstatin A, 20 µm phenylmethanesulphonyl fluoride [PMSF]). Briefly, extracts were centrifuged at 8,000 rpm for 20 min at 4°C. Supernatants were then centrifuged at 47,000 rpm for 30 min at 4°C and resulting supernatants were finally centrifuged at 47,000 rpm for 2.5 h at 4°C. The resulting protein pellets were resuspended in 150 µL of a buffer containing 20% glycerol, 5 mm MgCl_2_ and 50 mm Tris-Cl (pH 7.5) (Buffer A). Protein concentration was determined from these resuspended pellets (BIORAD® assay). The peptidase activities of the proteasome (chymotrypsin-like and trypsin-like activities) were determined by measuring the hydrolysis of the fluorogenic substrates succinyl-Leu-Leu-Val-Tyr-7-amido-4-methylcoumarin (LLVY-AMC) (Sigma, USA) and the Boc-Leu-Arg-Arg-7-amido-4-methylcoumarin (LRR-AMC) (Enzo Life Sciences) respectively. To measure the proteasome chymotrypsin-like and trypsin-like activities, 15 µg of proteins from the resuspended pellets diluted in 15 µL of Buffer A were added to 60 µl of medium containing 50 mm Tris-Cl (pH 7.5), 11.25 mm MgCL_2_, 1.25 mm DTT, 0.01 U apyrase and 300 µm LLVY-AMC or 800 µm LRR-AMC. Pilot experiments were performed with or without inhibitors of the chymotrypsin-like activity (MG132, Affiniti) to ensure that the activities were totally inhibited. The trypsin-like activity was measured with and without the specific inhibitor lactacystin (lactacystin, 100 µm, Sigma). Both activities were determined by measuring the accumulation of the fluorogenic cleavage product (AMC) using a fluorescence spectrometer FLX800 (Biotek, USA) during 45 min at 380 nm excitation wavelength and 440 nm emission wavelength. The time course for the accumulation of AMC after hydrolysis of the substrate was analyzed by linear regression to calculate activities, i.e., the slopes of best fit of accumulation AMC *vs.* time.

### Analysis of muscle protein synthesis and proteolysis signalling pathways by western blotting

Powder (300 mg) of *gastrocnemius* muscles was homogenized in 10 volumes of a buffer containing 1 mm dithiothreitol (DTT), 0.1 mm phenylmethanesulphonyl fluoride (PMSF), 1 mm benzamidine and 0.5 mm Na vanadate. Extracts were then centrifuged at 9,500 rpm for 12 min at 4°C. Aliquots of supernatants were diluted in sample buffer, boiled for 5 min, and stored at −20°C until protein immunoblot analyses. Equal amounts of proteins were separated by SDS–PAGE and transferred to PVDF membranes (GE Healthcare, Orsay, France).

#### Muscle proteasome proteolysis pathway

To explore the first step of the ubiquitin-proteasome-dependant proteolysis (tagging of proteins by ubiquitin before their recognition by the proteasome) the anti-ubiquitinylated proteins antibody, which recognizes poly-ubiquitin chains (Millipore, USA), was used at 1∶2,000 dilution. Twenty-five micrograms of proteins was separated on 7% acrylamide gels. FoxO3a is a transcription factor involved in atrogenes transcription. When phosphorylated, its nuclear translocation is impossible. Thus, the ratio between FOXO3a and its phosphorylated form traduces an inhibition of atrogenes transcription. The abundance of transcription factor FoxO3a and its phosphorylated form phospho-FoxO3a (Ser253) were determined using appropriate antibodies (Cell Signalling Technology, Inc., Danvers, MA, USA) at 1∶1,000. Fifty micrograms of proteins was separated on 7.5% acrylamide gels.

#### Muscle protein synthesis pathway

We have previously shown that mTOR signalling pathway in old rat muscles was activated after amino acids and glucose intake [31]–[32]. However, the early steps in this signalling pathways (S6K1) were only stimulated transiently (30 min) and were not anymore phosphorylated after 1 h. By contrast, the phosphorylation of the substrates of S6K1 such as S6 remained elevated even 1 h after the nutrient intake. Because, muscle protein synthesis was measured 110–140 min after food intake in the present study, only the downstream signalling factors of the S6K1 were assessed in order to reflect the activation of the mTOR pathway. Immunoblotting was performed using appropriate antibodies: S6 and phospho-S6 (Ser 235/236) (Cell Signalling Technology, Inc., Danvers, MA, USA) at 1∶6,000 dilution. To determine the amount of S6 25 µg of proteins were separated on 15% acrylamide gels, and 30 µg of proteins were separated on 12% acrylamide gels to quantify amount of phospho-S6. The amount of total 4EBP1 (α, β and γ forms) was determined on 50 µg of proteins separated on 15% acrylamide gels, using an antibody (Cell Signalling Technology, Inc., Danvers, MA, USA) at 1∶4,000 dilution.

For all immunoblots, signals were detected using the ECL+ detection kit (GE Healthcare, France) after exposition onto radiographic film (Hyperfilm ECL, GE Healthcare, France), quantified by densitometry using the Image J software and normalized against the amount of proteins (determined following Ponceau Red staining) to correct for uneven loading.

### Measurements of in vivo protein synthesis

Protein synthesis rates were measured using the flooding-dose method. Each rat was injected intravenously with [1–^13^C] valine (99%) (150 µmoles/100 g body weight), 40 min before sacrificing (i.e., 110–140 min after the beginning of the experimental diets), to flood the precursor pool with [1-^13^C] valine. Rats were then euthanized under pentobarbital sodium anaesthesia (50 mg kg^−1^ ip). Blood was withdrawn from the aorta, and hindlimb *gastrocnemius* muscles were carefully dissected, weighed and frozen in liquid nitrogen. Free and bound valine enrichments were determined as follows.

Muscles were powdered in liquid nitrogen in a ball mill (Dangoumeau, Prolabo, Paris, France). A 200 mg-aliquot of frozen muscle powder was homogenized in 2 mL of 10% trichloroacetic acid (TCA). Homogenates were centrifuged (8000 rpm, 15 min, 4°C) and supernatants, containing free amino acids, were desalted by cation-exchange chromotography (AG 50×8, 100–200 mesh, H^+^ form, Bio-Rad, Richmond, CA) in minidisposal columns. Valine and other amino acids were eluted with 4 mol/L NH_4_OH. After evaporation of NH_4_OH under vacuum, free amino acids were resuspended in 0.01 mol/L HCl for enrichment measurements. TCA-insoluble materials were washed 3 times in 4 volumes of cold 10% TCA and once in 4 volumes of 0.2 mol/L perchloric acid (PCA). Resultant pellets were resuspended in 0.3 mol/L NaOH and incubated at 37°C for 1 h. Protein concentration was determined using the bicinchoninic procedure. Proteins were precipitated with 20% PCA overnight at 4°C, samples centrifuged (10,000×*g*, 5 min, 4°C). The protein pellet was hydrolyzed in 6 mol/L HCl at 110°C for 24 h. HCl was removed by evaporation and amino acids purified by cation-exchange chromotography as described above. Measurement of free valine enrichment was done as its *t*-butyldimethylsilyl derivative by gas chromatography electron impact mass spectrometry, using a gas chromatograph coupled to an organic mass spectrometer quadrupole. Enrichment of [1-^13^C] valine into muscle proteins was measured as its *N*-acetyl-propyl derivatives by gas chromatography–combustion-isotope ratio mass spectrometry.

### Calculations

The absolute synthesis rate (ASR) was calculated from the product of the protein fractional synthesis rate (FSR) and the protein content of the tissue and expressed in mg/d. FSR (in %/d) is calculated from the formula : FSR = S*b*×100/S*a*×*t*, were S*b* is the enrichment at time t (minus natural basal enrichment of protein) of the protein-bound valine, *t* is the incorporation time in d, and S*a* is the mean enrichment of free tissues valine between time 0 and *t*. The mean S*a* enrichment was the S*a* (t_1/2_) value calculated from the linear regression obtained in tissue between time 0 and time *t*.

### Statistical analysis

All data are expressed as means ± SEM. Food intake and body weight comparisons were assessed using repeated measures analysis of variance test (StatView statistical software package, version 5, SAS Institute, Cary, NC, USA). Other measurements were analyzed using a two-way ANOVA (time, diet). When significant differences were detected by ANOVA, *post hoc* comparisons between groups were made using the Fisher's PLSD test. Significance was defined at the P<0.05 level.

## Results

### Body weights and food intake

**Figure 1:**
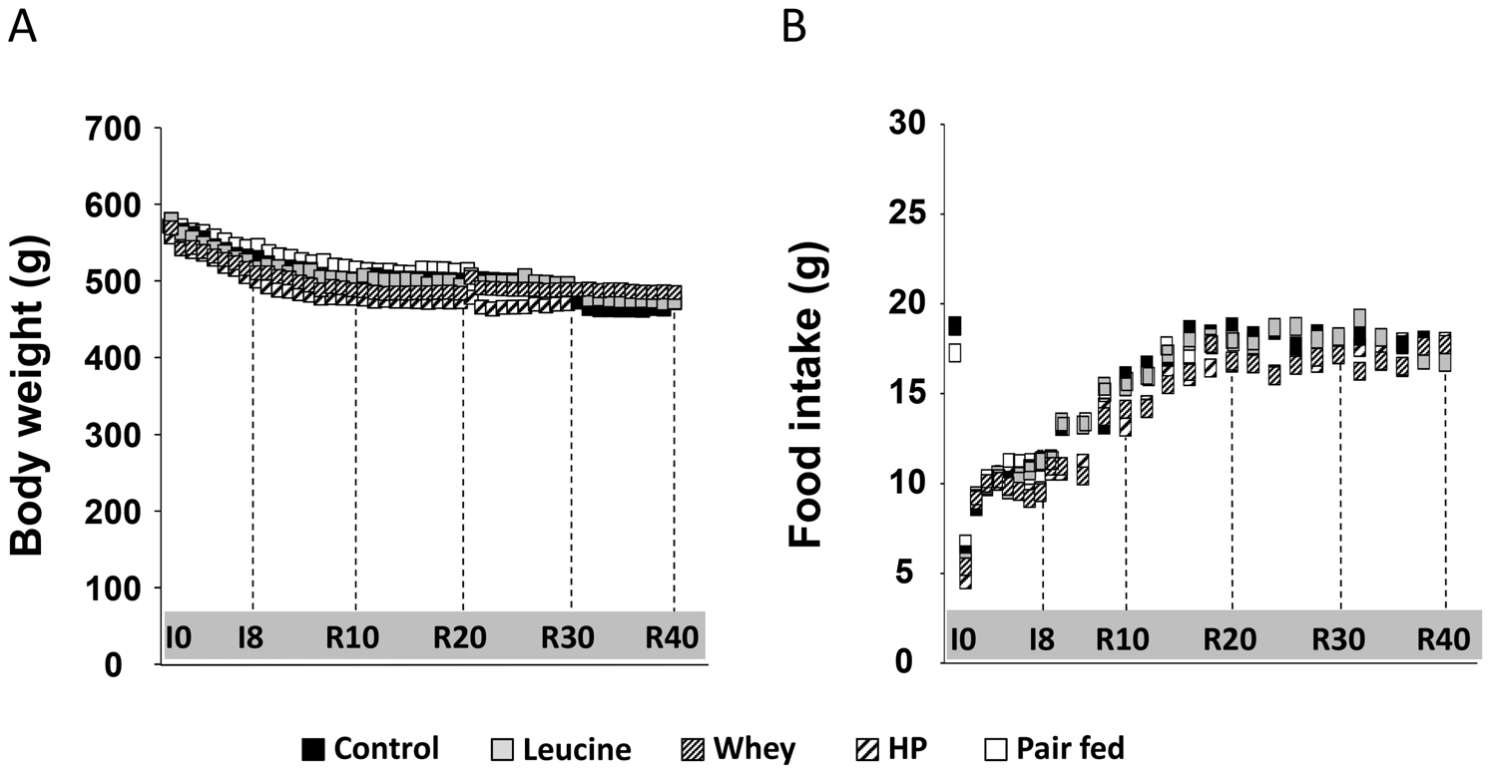
Body weight and food intake for rats fed the CONTROL, LEU, WHEY, and HIGH PROT diets. (*A*), the initial body weight for rats (expressed in g) were similar between the four experimental groups at I0 (Experiment 1 and Experiment 2). Changes were similar across the groups during the whole experiment, i.e. casting induced a decreased of body weight which stabilized during the recovery phase. (*B*), food intake (expressed in g.day^−1^) was similar between groups from the beginning to the end of the experiment. LEU, leucine; WHEY, whey protein; HIGH PROT, high protein. I0, before immobilization; I8, 8 days of casting; R10 to R40, 10 to 40 days of recovery.

Body weight and food intake are presented in **Fig. 1**. Body weight was similar between the CONTROL, LEU, WHEY and HIGH PROT groups at I0 (560–580 g) and had the same body weight decrease in response to casting (−10% at I8) (**Fig. 1A**). During the recovery period, body weights stabilized in all groups by the end of the recovery period (∼470 g at R40). Similarly, food intake decreased until I8 in the four experimental groups, and then increased to reach normal value (16–17 g.day^−1^ at R40 *vs.* 18 g.day^−1^ at I0) (**Fig. 1B**). Food intake of the pair-fed group perfectly matched that of the casted group during immobilization (11.29±0.06 g.day^−1^ at I8) and recovery (17.86±1.09 g.day^−1^ at R20). The body weight of pair-fed animals decreased slightly during the experimental protocol (−4.4% at I8 and 10.1% at R20)

### Effect of the nutritional intervention on muscle mass

**Figure 2:**
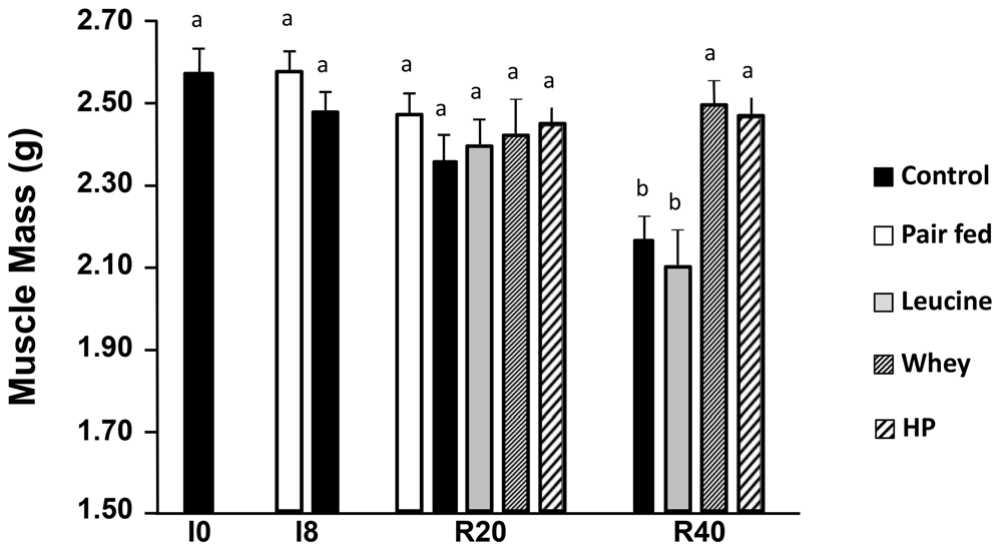
Muscle mass of the non-immobilized *gastrocnemius* muscles for rats fed the CONTROL, LEU, WHEY, and HIGH PROT diets. Muscle mass is expressed for each group in grams (g). Muscle mass slightly decreased when rats were fed the CONTROL and LEU diets to become significantly lower at R40 (Experiment 1). However, muscle mass was maintained at its initial level in rats fed the WHEY and HIGH PROT diets (Experiment 2). LEU, leucine; WHEY, whey protein; HIGH PROT, high protein; I0, before immobilization; I8, 8 days of casting; R20 and R40, 20 and 40 days of recovery. Values with different letters are significantly different from each other. Data are means ± SEM.

*Gastrocnemius* muscle mass from pair-fed non-immobilized old adult rats was stable at each point measured (2.553±0.04; 2.557±0.03 and 2.498±0.04 g at I0; I8 and R20, respectively) (**Fig. 2**). This result confirmed our previous observations [5] showing that pair-fed *gastrocnemius* weight did not significantly change from I0 to R40.

In controls, *gastrocnemius* muscle mass of the non-immobilized limb remained unchanged during the immobilization (I8) and the first 20 days of recovery (**Fig. 2**). Between the 20^th^ and the 40^th^ day of recovery *gastrocnemius* atrophied significantly by 15.5%. By comparison, the immobilized *gastrocnemius* mass of the same animals was decreased by 20% at R40 [28]. The atrophy in other muscles has also been checked for the tibialis anterior. In the non-immobilized leg, tibialis anterior in casted animals was also atrophied significantly at R40 compared to before immobilization (I0) (0.79±0.02 vs. 0.89±0.02 mg (−11%; P<0.05) at R40 and I0, respectively) whereas it remained unchanged just after the immobilization period (I8 = 0.90±0.03 mg). According to these data, tibialis anterior followed the same weight change than the gastrocnemius muscle in the non-immobilized limb in casted animals.

When rats were fed the leucine supplemented diet, *gastrocnemius* muscle followed the same kinetic of atrophy (not significant at R20 and −18.1% (P<0.05) at R40. However, when fed the whey protein or the high protein diets, *gastrocnemius* muscle of the non-immobilized limb remained stable throughout the experimental period and did not differ in mass when compared to I0 (**Fig. 2**).

### Ubiquitin-proteasome-dependent pathway in the non-immobilized leg of casted animals

**Figure 3:**
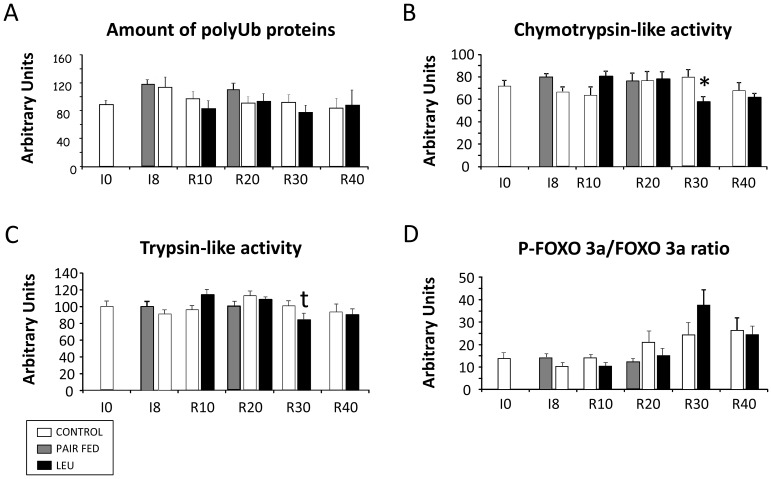
Ubiquitin–proteasome-dependent proteolysis in the non-immobilized *gastrocnemius* muscles of rats fed the CONTROL and LEU diets. Ubiquitin proteasome-dependent proteolysis was measured in Experiment 1. As no effect of the meal was observed, post-absortive and post-prandial values are pooled. (*A*), accumulation of polyUb proteins was assessed on 25 µg of proteins by immunoblotting using an antibody that recognizes polyubiquitin chains. (*B*) and (*C*), the chymotrypsin-like activity (*B*) and trypsin-like activity (*C*) of the proteasome were measured by using the fluorogenic substrate succinyl-LLVY-AMC and Boc-LRR-AMC as indicated in Methods. Data are expressed in relative fluorescence units (RFU µg−1 min−1). (*D*), phospho-FoxO3a/FoxO3a ratio traduces an anti-proteolytic potential. FoxO3a and its phosphorylated form phospho-FoxO3a (Ser253) were determined using appropriate antibodies on 50 µg of proteins. LEU, leucine; PolyUb, polyubiquitinated; I0, before immobilization; I8, 8 days of casting; R10 to R40, 10 to 40 days of recovery. * *P*<0.05, *vs.* I0. Data are means ± SEM.

No difference was observed between the PA and the PP states. Values from both groups were thus pooled. **Fig. 3A** shows that neither immobilization of the controlateral leg, nor the diet consumed during the recovery period affected significantly the amount of polyUb conjugates in the non-immobilized limb. Both chrymotrypsin- (**Fig. 3B**) and trypsin-like activities (**Fig. 3C**) were unaffected and no difference was observed between the CONTROL group and the LEU group during the whole experiment. Phospho-FOXO3a/FOXO3a ratio was not different between the two diets (**Fig. 3D**). All values were not significantly different from the pair fed values (data not shown). As previously recorded, an increase of muscle proteolysis during the recovery period could not explain the decrease of muscle mass of the non-immobilized limb during the rehabilitation period [28]. Furthermore, leucine supplementation had no effect on proteolysis showing that nutritional intervention on muscle proteolysis would be unlikely efficient.

### Effect of the nutritional interventions on the in vivo post-prandial muscle protein synthesis

**Figure 4:**
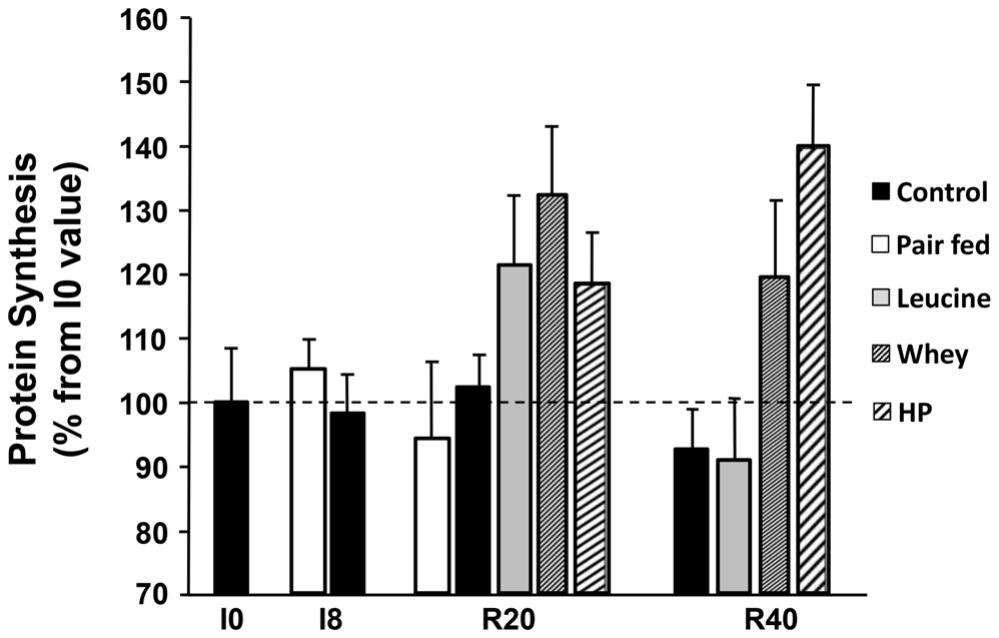
*In vivo* post-prandial protein synthesis in non-immobilized *gastrocnemius* muscles of old adult rats fed the CONTROL, LEU, WHEY, and HIGH PROT diets. *In vivo* protein synthesis is expressed for each group in % of I0 at I8 (end of immobilization period) and R40 (end of recovery period, i.e. after 40 days of dietary supplementation) for Experiment 1 (Control and LEU groups) and Experiment 2 (WHEY and HIGH PROT groups). LEU, leucine; WHEY, whey protein; HIGH PROT, high protein; I0, before immobilization; I8, 8 days of casting; R40, 40 days of recovery. Values with different letters are significantly different from each other. Data are means ± SEM.

Muscle protein synthesis stimulation is the other driver of protein metabolism especially after food intake and when a nutritional strategy is studied. When rats were fed a control diet, muscle post-prandial protein synthesis in the non-immobilized limb remained unchanged during the immobilization period (I0 *vs.* I8) and during the recovery period (R20 and R40 *vs.* I0) (**Fig. 4**). With the leucine supplemented diet, muscle protein synthesis was higher than with the control diet at R20 but returned to control values at R40. With the whey protein and high protein diets, post-prandial muscle protein synthesis followed the same pattern than the leucine-supplemented group but remained significantly higher at R40 when compared to the control and the leucine supplemented groups (**Fig. 4**).

### Muscle protein synthesis kinetics at the post-absorptive and the post-prandial states in the pair-fed, control and leucine-supplemented old adult rats

**Figure 5:**
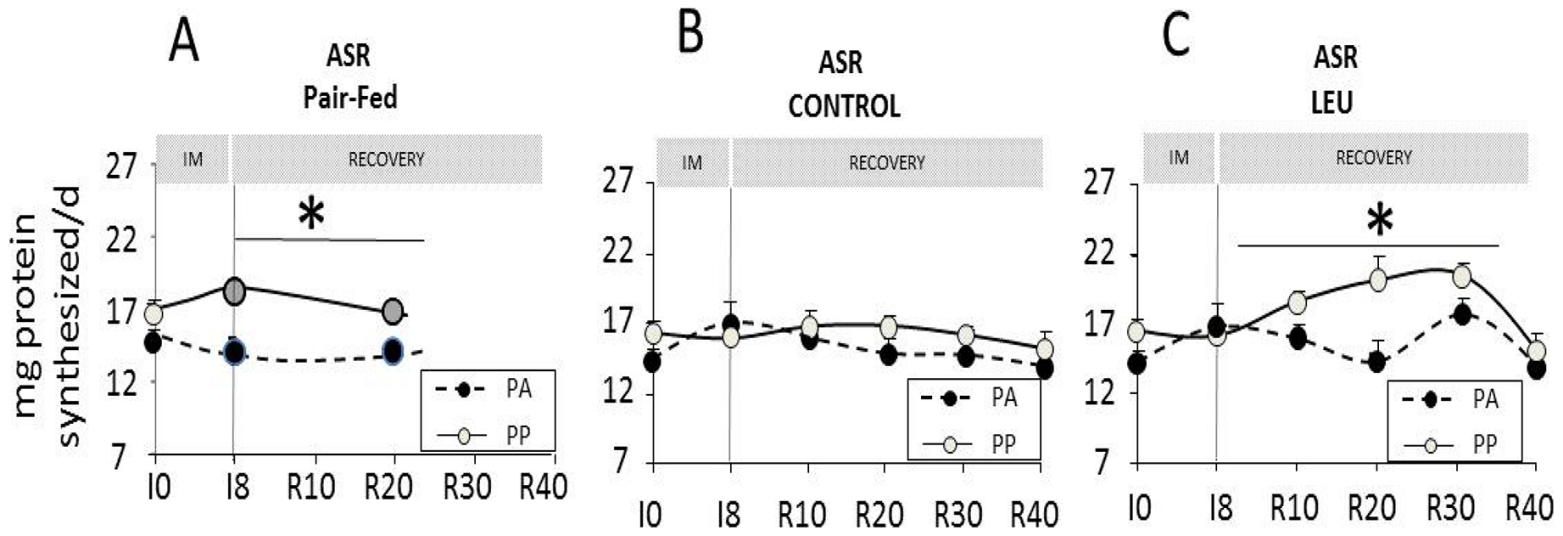
Kinetic protein synthesis measurement in non-immobilized *gastrocnemius* muscles of PAIR-FED, CONTROL and LEU old adult rats. Muscle protein synthesis at I0 and I8 in the post-absorptive and the post-prandial states for each time is expressed as the absolute synthesis rate (ASR), i.e. the amount of proteins synthesized in mg.day^−1^ for rat fed the pair-fed (*A*), CONTROL (*B*) and LEU (*C*) diets. LEU, leucine; IM, immobilization period; I0, before immobilization; I8, 8 days of casting; R10 to R40, 10 to 40 days of recovery; PA, post-absorptive state; PP, post-prandial state. * *P*<0.05, PA *vs.* PP. Values with different letters are significantly different from each other. Data are means ± SEM.

At I0, muscle protein synthesis was slightly increased after food intake but the difference remained non significant (**Fig. 5**). This reflects, as previously described, the anabolic resistance observed during ageing. In the non-immobilized pair-fed group, the difference between the PA and PP protein synthesis became significant at I8 and R20 and reached +29.3% and +19.4%. (**Fig. 5A**). By contrast, in the control group, during the immobilization and the recovery period, PA and PP protein synthesis remained unchanged in the non-immobilized limb and still exhibited an anabolic resistance (**Fig. 5B**). When animals were fed the leucine-supplemented diet, muscle protein synthesis at the PP state became significantly different from the PA states from R10 to R30. At R40, no significant difference was again recorded (**Fig. 5C**).

**Figure 6:**
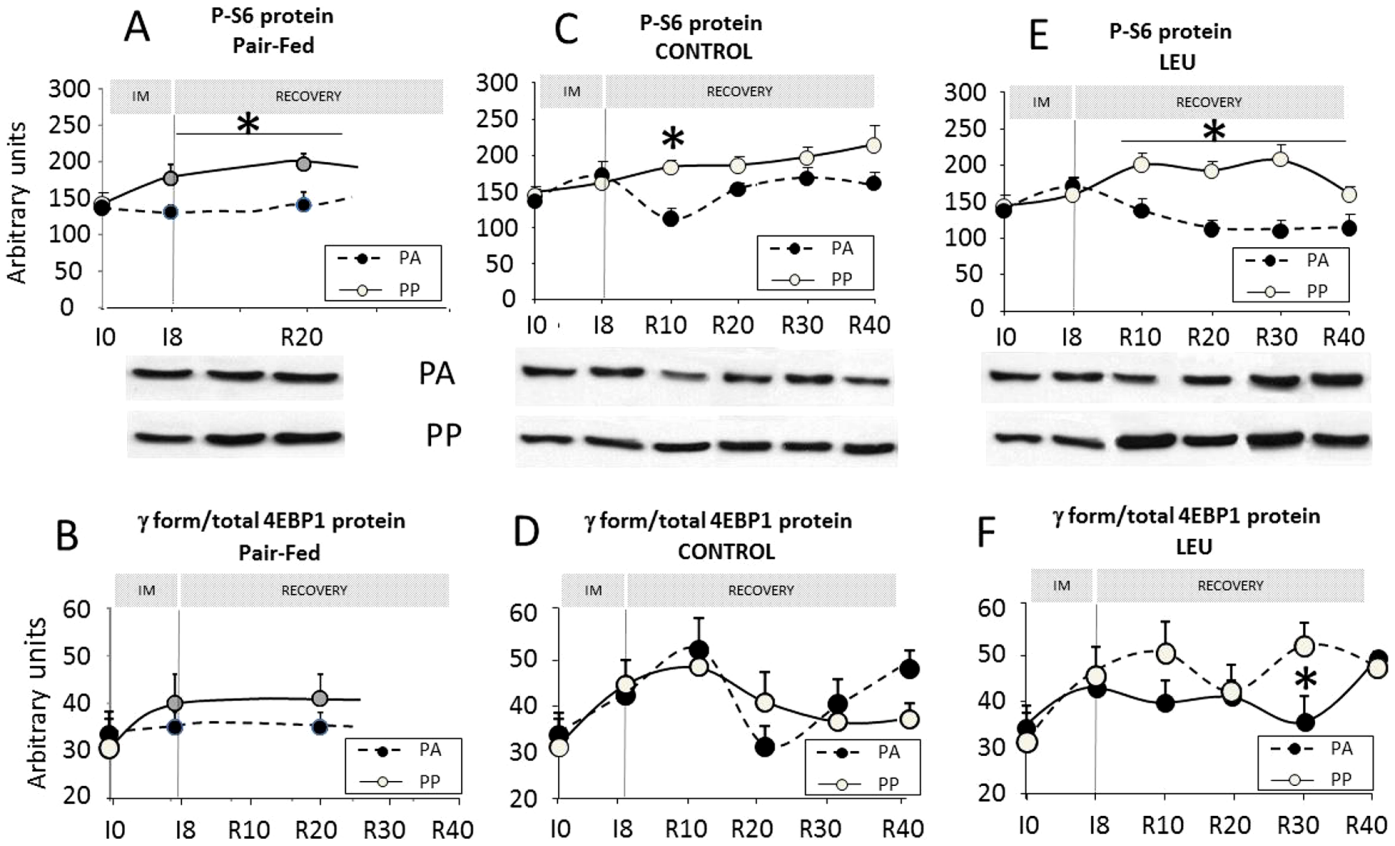
Muscle protein synthesis signalling pathway in *gastrocnemius* muscles of pair-fed rats and in the non-immobilized *gastrocnemius* muscles of old adult rats fed the CONTROL and LEU diets. Muscle protein synthesis signalling pathway was measured in Experiment 1. Protein S6 phosphorylation in *gastrocnemius* is expressed in arbitrary units for the pair-fed (*A*), CONROL (*C*) and the LEU groups (*E*) in the post-absorptive and the post-prandial states. Representative immunoblots are also shown. The amount of protein S6 phosphorylated was assessed by immunoblotting on 30 µg of proteins. Amount of protein 4EBP1 is expressed in arbitrary units as the ratio *γ* form/total forms in the pair-fed (*B*), CONROL (*D*) and the LEU groups (*F*). LEU, leucine; IM, immobilization period; I0, before immobilization; I8, 8 days of casting; R10 to R40, 10 to 40 days of recovery; PA, post-absorptive state; PP, post-prandial state. * *P*<0.05, PA *vs.* PP. Data are means ± SEM.

Within the mTOR signalling pathways, the phosphorylation of the S6 protein did not change with food intake at I0 (**Fig. 6**). In the non-immobilized pair-fed group, the phosphorylation of the S6 protein become significantly higher in the PP then in the PA state in response to the food decrease at both I8 (+34%) and R20 (+39%)(**Fig. 6A**). By contrast, in the CONTROL group, during the immobilization and the recovery period, no significant increase in the S6 protein phosphorylation was recorded in the non-immobilized limb in response to food intake (excepted at R10) (**Fig. 6C**). When animals were fed the leucine-supplemented diet, the phosphorylation of S6 greatly increased after food intake in the non-immobilized limb (+61%, +54% and +91% at R10, R20 and R30, respectively, PA *vs.* PP) (**Fig. 6E**). At R40, no significant stimulation was recorded.

Regarding the phosphorylation of 4EBP1, no difference between the PA and PP states was recorded either during the immobilization period or the recovery period in all groups (**Fig. 6B,D,F**) excepted at R30 in the leucine supplemented group (**Fig. 6F**).

### Correlation analysis between muscle protein synthesis and muscle protein content

**Figure 7:**
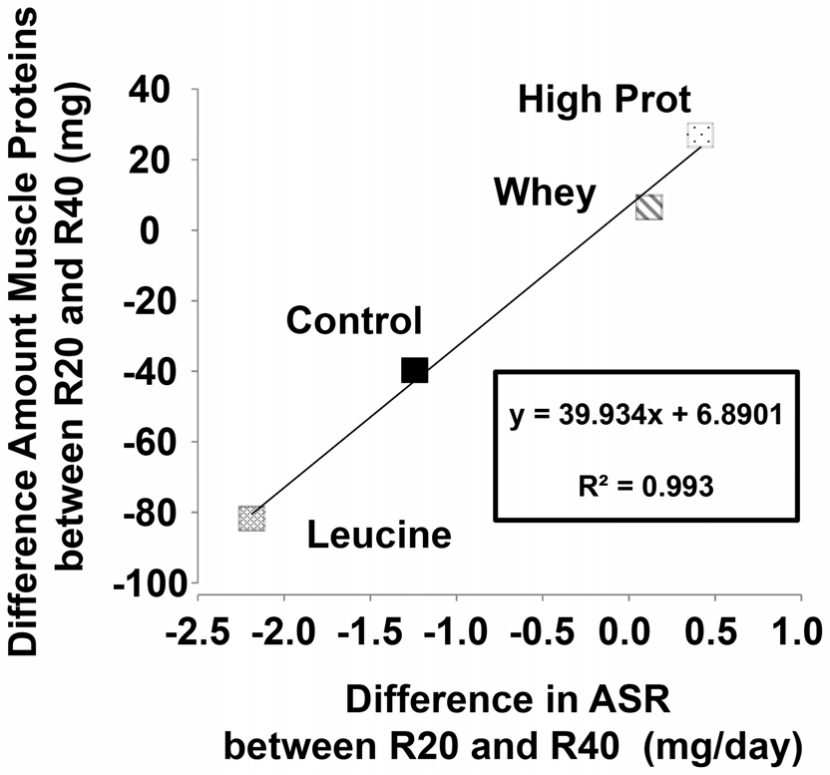
Relationship between post-prandial muscle protein synthesis and muscle protein content during muscle mass loss occurring during recovery. The difference in total Absolute Synthesis Rate (ASR) expressed in mg/day was calculated between R20 and R40, i.e. when muscle atrophied, and correlated to the muscle protein content in Experiment 1 (Control and Leucine groups) and Experiment 2 (Whey and High Prot groups).

The Absolute Synthesis Rate (ASR) was calculated in mg/day, i.e. the amount of proteins synthesized each day. Between R20 and R40, i.e. when muscles atrophied, total ASR was positively correlated with the amount of proteins measured into the gastrocnemius muscles across all groups (**Fig. 7**, R^2^ = 0.993), demonstrating that the efficiency of protein synthesis was correlated with the muscle protein content. With leucine and control diets, this correlation was negative i.e. fewer proteins were synthesized at R40 when compared with R20 (−80 and −40 mg for Leucine and Control groups, respectively). With whey and high protein diets, the correlation was positive, that i.e. at R40 as much or more proteins were synthesized *vs.* R20.

## Discussion

We demonstrated that a simple adverse event (i.e. a relatively short period of immobilization) without any associated pathology and which is localized only to one limb, has deleterious consequences by inducing a delayed generalized muscle loss in old adult rats. Such situations may be associated to the geriatric frailty syndrome that places older individuals at high risk for adverse outcomes such as falls, disability, morbidity and institutionalization [7]–[10].

More precisely, in this study, unilateral hindlimb casting in old adult rats induced a muscle mass loss of the controlateral non-immobilized leg 20 days after cast removal when animals were fed a standard protein diet (i.e. 13% casein as protein source) [5]. It is important to note that this atrophy was similar to the atrophy observed in the immobilized limb which reached 20% in the same rats [28]. However, it could not be attributed to the ageing processes *per se* because the non-immobilized pair-fed old adult rats of the same age did not present this atrophy in the present experiment and as previously observed [5]. As muscle protein breakdown was unchanged in the non-immobilized leg of casted rats in the present study, our working hypothesis was that protein synthesis was altered. Before immobilization, old adult rat muscles presented the well-known anabolic resistance [33] associated with the impaired response of the mTOR signalling pathway which was activated post-prandially [34]. However, interestingly, in the non-immobilized pair-fed group, muscle protein synthesis sensitivity to food intake was partially restored when animals were subjected to food restriction. It can be explained by the restoration of the activation of the mTOR signalling pathway since we recorded simultaneously a significant stimulation of the S6 protein following food intake. Even though we may not had enough data and despite the fact that experiment was not designed to answer this question, we may nevertheless hypothesize that old adult rats can still adapt to moderate food deprivation and can partially restore a post-prandial stimulation of muscle protein synthesis when the dietary supply in amino acids decreases. This adaptation may in part explain why the non-immobilized old adult pair-fed rats were able to constantly maintain their muscle mass in the present study. By contrast, we showed here that unilateral hindlimb casting did not modify muscle protein synthesis at both the post-absorptive and the post-prandial states in the non-immobilized limb when animals were fed the control diet and when these values were compared to those recorded at I0 (before casting). In other words, immobilization prevented the restoration of muscle protein synthesis sensitivity to food intake as observed in the pair-fed group and animals remained in an anabolic resistance state. This non-adaptation, caused by immobilization in the non-immobilized limb may explain the decrease in muscle mass recorded in this group because the post-prandial muscle protein synthesis could not adapt to the decrease of amino acid supply. The mechanisms implicated remained unknown and would need further investigation. We may postulate that, at the time we measured muscle protein synthesis (2.–2.5 h post-prandially), no difference in the immobilized group could be seen because the overall post-prandial stimulation of protein synthesis was less efficient and/or not sustained enough when compared to the old adult pair-fed group. Indeed, a stimulation of the post-prandial muscle protein synthesis in the non-immobilized limb could not be excluded but it may have been terminated before the sacrifice of the animals. The maintenance of the anabolic resistance in the non-immobilized limb could not be attributed to the development of an inflammatory or/and oxidative stress which could not be detected during the immobilization or the recovery period [5].

The anabolic resistance of muscle protein synthesis could be overcome with adapted nutritional strategies [35]. We are aware that physical activity is the best way to maintain and/or gain muscle mass. In elderly individuals, resistance exercise increased muscle protein synthesis by 30 to 50% [36]–[38]. However, this countermeasure is not always applicable particularly in the elderly population when immobilization results from a trauma, and therefore others solutions should be proposed. Amino acids are able to stimulate muscle anabolism [39], [40] by increasing protein synthesis and decreasing protein degradation [41]–[46]. As leucine is an amino acid trigger that has been shown to stimulate by itself protein synthesis and decrease protein breakdown *in vitro* [15], [20], [23] and *in vivo* [12], [13], [17], [19], [22], we expected that a free leucine-supplemented diet could improve protein metabolism and therefore prevent and/or limit the general muscle mass loss we observed following local immobilization without bed rest. We found here that free leucine was able to better stimulate protein synthesis as previously described in the literature [16], [17], [22], [30]. This increase of post-prandial protein synthesis correlated with an increase of the mTOR signalling pathway, i.e. an increased phosphorylation of S6 protein when compared to the control non supplemented diet. However, this improvement in protein synthesis rate did not prevent the general muscle atrophy observed during the rehabilitation period following unilateral hindlimb casting.

Once again, this implies that the stimulatory effect of leucine in the non-immobilized leg may have been weak or may not have been adequately sustained to induce the sufficient positive nitrogen balance required to maintain muscle mass [35]. Verhoven et al. [47] and Zeanandin et al. [31] used long-term leucine supplementation in healthy elderly individuals or in the animal model and did not show any beneficial effect on muscle mass. This discrepancy may be explained by a desynchronization between the leucine stimulatory signal (which may be transient) and the slow and late liberation of amino acids (coming from casein digestion which is slowly digested) [28], [35], [48]–[50]. Choosing free leucine as a supplement over a normal protein diet creates a de-synchronization between leucine signal and the rise in all amino acids. Indeed the free leucine is absorbed immediately whereas the other amino acids are released later after gastric emptying and proteolytic digestion in the gut. This non synchronization between the stimulation of muscle leucine-associated protein metabolism pathways and the delayed availability of amino acids as substrates can explain that protein anabolism was only stimulated for a very short period of time during the postprandial period and then could not translate into a significant muscle protein accretion [35].

To induce a sustained stimulation of muscle protein synthesis during the post-prandial phase within the non-immobilized limb, we tested whey proteins and a high protein diet. This nutritional strategy has been shown to be a lot more efficient than free leucine supplementation in the immobilized limb and was able not only to re-stimulate muscle protein synthesis post-prandially but also to generate a significant muscle mass recovery [28]. Because whey proteins are leucine-rich- and fast-digested proteins, not only could the post-prandial aminoacidemia be improved but also, leucine availability which is increased simultaneously with the other amino acids. High protein diets are also an efficient strategy to generate hyperaminoacidemia but they present the advantage to also prolong the hyper aminoacidemia during the post-prandial period [51]. A prolonged stimulation could not be achieved with fast-digested proteins at normal dietary level (even enriched with leucine) since the concentration of amino acids declines rapidly after their intake [48]. We showed here that both diets had a positive impact on the non-immobilized muscle mass and prevented the general muscle atrophy. These two diets have induced a greater post-prandial protein synthesis stimulation compared to casein or free leucine-supplemented diets especially in the late phase of recovery, i.e. when the atrophy of the controlateral limb appeared. The hypothesis is that both diets (the whey protein and high protein diets), simultaneously provided the signal (leucine) and the overall amino acids supply necessary to induce higher and longer amino acid concentrations capable to prolong muscle protein synthesis within the skeletal muscle. Since both diets are rich in leucine, this effect could also been associated (concomitantly or not) to an enhanced post-prandial hyperinsulinemia. This hypothesis seems unlikely since whey protein ingestion and a high protein diet are associated with a lower glycemic response [52]–[54]. In order to test the relationship between preserved nitrogen balance and a sustained post-prandial anabolism in muscle, a post-hoc correlation analysis was performed on the values of muscle protein synthesis obtained for all groups during the time period from R20 to R40 where there is significant muscle mass loss. This demonstrated that during this period, muscle protein synthesis was sustained (i.e. the amount of protein synthesized was similar at R20 and R40) and correlated with an increased muscle protein content, which was not the case with the leucine and control diets. These results tend to confirm that both diets were able to sustain the positive nitrogen balance required to maintain the muscle mass constant. However it remains unknown if this effect only and specifically involves an enhanced post-prandial anabolism and further investigations is needed to determine if these diets sustain the nitrogen balance by specifically maintaining the muscle protein sensitivity to food intake or also at the post-absorptive state.

The present findings show that nutrition is able to play a crucial role in the maintenance of whole body muscle mass when old adult rats are in rehabilitation after a catabolic state even if the atrophy is localized to one limb without bed rest. Indeed, we have demonstrated that after unilateral hindlimb immobilization, a significant atrophy appeared in non-immobilized hindlimb when old adult rats were fed a ‘classic’ diet containing casein as protein source. Furthermore, we highlighted that a specific nutrition (e.g. whey protein and high protein diets) was efficient to prevent this global adverse effect following a local cast immobilization.

## References

[1] Cruz-Jentoft AJ, Baeyens JP, Bauer JM, Boirie Y, Cederholm T, et al (2010) Sarcopenia: European consensus on definition and diagnosis: Report of the European Working Group on Sarcopenia in Older People. Age Ageing 39: 412–423 afq034 [pii];10.1093/ageing/afq034 [doi]. 20392703 10.1093/ageing/afq034 PMC2886201

[2] English KL, Paddon-Jones D (2010) Protecting muscle mass and function in older adults during bed rest. Curr Opin Clin Nutr Metab Care 13: 34–39 10.1097/MCO.0b013e328333aa66 [doi]. 19898232 10.1097/MCO.0b013e328333aa66 PMC3276215

[3] Mosoni L, Malmezat T, Valluy MC, Houlier ML, Attaix D, et al (1999) Lower recovery of muscle protein lost during starvation in old rats despite a stimulation of protein synthesis. Am J Physiol 277: E608–E616. 10516119 10.1152/ajpendo.1999.277.4.E608

[4] Dardevet D, Sornet C, Taillandier D, Savary I, Attaix D, et al (1995) Sensitivity and protein turnover response to glucocorticoids are different in skeletal muscle from adult and old rats. Lack of regulation of the ubiquitin-proteasome proteolytic pathway in aging. J Clin Invest 96: 2113–2119 10.1172/JCI118264 [doi]. 7593595 10.1172/JCI118264 PMC185859

[5] Magne H, Savary-Auzeloux I, Vazeille E, Claustre A, Attaix D, et al (2011) Lack of muscle recovery after immobilization in old rats does not result from a defect in normalization of the ubiquitin-proteasome and the caspase-dependent apoptotic pathways. J Physiol 589: 511–524 jphysiol.2010.201707 [pii];10.1113/jphysiol.2010.201707 [doi]. 21115641 10.1113/jphysiol.2010.201707 PMC3055540

[6] Vazeille E, Codran A, Claustre A, Averous J, Listrat A, et al (2008) The ubiquitin-proteasome and the mitochondria-associated apoptotic pathways are sequentially downregulated during recovery after immobilization-induced muscle atrophy. Am J Physiol Endocrinol Metab 295: E1181–E1190 90532.2008 [pii];10.1152/ajpendo.90532.2008 [doi]. 18812460 10.1152/ajpendo.90532.2008

[7] Dayhoff NE, Suhrheinrich J, Wigglesworth J, Topp R, Moore S (1998) Balance and muscle strength as predictors of frailty among older adults. J Gerontol Nurs 24: 18–27. 9801527 10.3928/0098-9134-19980701-06

[8] Ferrucci L, Cavazzini C, Corsi A, Bartali B, Russo CR, et al (2002) Biomarkers of frailty in older persons. J Endocrinol Invest 25: 10–15. 12508906

[9] Fried LP, Tangen CM, Walston J, Newman AB, Hirsch C, et al (2001) Frailty in older adults: evidence for a phenotype. J Gerontol A Biol Sci Med Sci 56: M146–M156. 11253156 10.1093/gerona/56.3.m146

[10] Walston J (2004) Frailty–the search for underlying causes. Sci Aging Knowledge Environ 2004: e4 10.1126/sageke.2004.4.pe4 [doi];2004/4/pe4 [pii]. 10.1126/sageke.2004.4.pe4 14749522

[11] Chen YW, Gregory CM, Scarborough MT, Shi R, Walter GA, et al (2007) Transcriptional pathways associated with skeletal muscle disuse atrophy in humans. Physiol Genomics 31: 510–520 00115.2006 [pii];10.1152/physiolgenomics.00115.2006 [doi]. 17804603 10.1152/physiolgenomics.00115.2006

[12] Anthony JC, Anthony TG, Layman DK (1999) Leucine supplementation enhances skeletal muscle recovery in rats following exercise. J Nutr 129: 1102–1106. 10356072 10.1093/jn/129.6.1102

[13] Anthony TG, Anthony JC, Yoshizawa F, Kimball SR, Jefferson LS (2001) Oral administration of leucine stimulates ribosomal protein mRNA translation but not global rates of protein synthesis in the liver of rats. J Nutr 131: 1171–1176. 11285321 10.1093/jn/131.4.1171

[14] Koopman R, Wagenmakers AJ, Manders RJ, Zorenc AH, Senden JM, et al (2005) Combined ingestion of protein and free leucine with carbohydrate increases postexercise muscle protein synthesis in vivo in male subjects. Am J Physiol Endocrinol Metab 288: E645–E653 00413.2004 [pii];10.1152/ajpendo.00413.2004 [doi]. 15562251 10.1152/ajpendo.00413.2004

[15] Buse MG, Reid SS (1975) Leucine. A possible regulator of protein turnover in muscle. J Clin Invest 56: 1250–1261 10.1172/JCI108201 [doi]. 1237498 10.1172/JCI108201 PMC301988

[16] Combaret L, Dardevet D, Rieu I, Pouch MN, Bechet D, et al (2005) A leucine-supplemented diet restores the defective postprandial inhibition of proteasome-dependent proteolysis in aged rat skeletal muscle. J Physiol 569: 489–499 jphysiol.2005.098004 [pii];10.1113/jphysiol.2005.098004 [doi]. 16195315 10.1113/jphysiol.2005.098004 PMC1464228

[17] Dardevet D, Sornet C, Bayle G, Prugnaud J, Pouyet C, et al (2002) Postprandial stimulation of muscle protein synthesis in old rats can be restored by a leucine-supplemented meal. J Nutr 132: 95–100. 11773514 10.1093/jn/132.1.95

[18] Frexes-Steed M, Lacy DB, Collins J, Abumrad NN (1992) Role of leucine and other amino acids in regulating protein metabolism in vivo. Am J Physiol 262: E925–E935. 1616025 10.1152/ajpendo.1992.262.6.E925

[19] Katsanos CS, Kobayashi H, Sheffield-Moore M, Aarsland A, Wolfe RR (2005) Aging is associated with diminished accretion of muscle proteins after the ingestion of a small bolus of essential amino acids. Am J Clin Nutr 82: 1065–1073 82/5/1065 [pii]. 16280440 10.1093/ajcn/82.5.1065

[20] Li JB, Jefferson LS (1978) Influence of amino acid availability on protein turnover in perfused skeletal muscle. Biochim Biophys Acta 544: 351–359 0304-4165(78)90103-4 [pii]. 719005 10.1016/0304-4165(78)90103-4

[21] Nakashima K, Ishida A, Yamazaki M, Abe H (2005) Leucine suppresses myofibrillar proteolysis by down-regulating ubiquitin-proteasome pathway in chick skeletal muscles. Biochem Biophys Res Commun 336: 660–666 S0006-291X(05)01858-9 [pii];10.1016/j.bbrc.2005.08.138 [doi]. 16153608 10.1016/j.bbrc.2005.08.138

[22] Rieu I, Balage M, Sornet C, Giraudet C, Pujos E, et al (2006) Leucine supplementation improves muscle protein synthesis in elderly men independently of hyperaminoacidaemia. J Physiol 575: 305–315 jphysiol.2006.110742 [pii];10.1113/jphysiol.2006.110742 [doi]. 16777941 10.1113/jphysiol.2006.110742 PMC1819434

[23] Smith K, Barua JM, Watt PW, Scrimgeour CM, Rennie MJ (1992) Flooding with L-[1-13C]leucine stimulates human muscle protein incorporation of continuously infused L-[1-13C]valine. Am J Physiol 262: E372–E376. 1550230 10.1152/ajpendo.1992.262.3.E372

[24] Tischler ME, Desautels M, Goldberg AL (1982) Does leucine, leucyl-tRNA, or some metabolite of leucine regulate protein synthesis and degradation in skeletal and cardiac muscle? J Biol Chem 257: 1613–1621. 6915936

[25] Anthony JC, Anthony TG, Kimball SR, Vary TC, Jefferson LS (2000) Orally administered leucine stimulates protein synthesis in skeletal muscle of postabsorptive rats in association with increased eIF4F formation. J Nutr 130: 139–145. 10720160 10.1093/jn/130.2.139

[26] Atherton PJ, Etheridge T, Watt PW, Wilkinson D, Selby A, et al (2010) Muscle full effect after oral protein: time-dependent concordance and discordance between human muscle protein synthesis and mTORC1 signaling. Am J Clin Nutr 92: 1080–1088 ajcn.2010.29819 [pii];10.3945/ajcn.2010.29819 [doi]. 20844073 10.3945/ajcn.2010.29819

[27] Peyrollier K, Hajduch E, Blair AS, Hyde R, Hundal HS (2000) L-leucine availability regulates phosphatidylinositol 3-kinase, p70 S6 kinase and glycogen synthase kinase-3 activity in L6 muscle cells: evidence for the involvement of the mammalian target of rapamycin (mTOR) pathway in the L-leucine-induced up-regulation of system A amino acid transport. Biochem J 350 Pt 2: 361–368. 10947949 PMC1221262

[28] Magne H, Savary-Auzeloux I, Migne C, Peyron MA, Combaret L, et al (2012) Contrarily to whey and high protein diets, dietary free leucine supplementation cannot reverse the lack of recovery of muscle mass after prolonged immobilization during ageing. J Physiol 590: 2035–2049 jphysiol.2011.226266 [pii];10.1113/jphysiol.2011.226266 [doi]. 22351629 10.1113/jphysiol.2011.226266 PMC3573319

[29] Reeves PG (1997) Components of the AIN-93 diets as improvements in the AIN-76A diet. J Nutr 127: 838S–841S. 9164249 10.1093/jn/127.5.838S

[30] Rieu I, Sornet C, Bayle G, Prugnaud J, Pouyet C, et al (2003) Leucine-supplemented meal feeding for ten days beneficially affects postprandial muscle protein synthesis in old rats. J Nutr 133: 1198–1205. 12672943 10.1093/jn/133.4.1198

[31] Zeanandin G, Balage M, Schneider SM, Dupont J, Hebuterne X, et al (2012) Differential effect of long-term leucine supplementation on skeletal muscle and adipose tissue in old rats: an insulin signaling pathway approach. Age (Dordr) 34: 371–387 10.1007/s11357-011-9246-0 [doi]. 21472380 10.1007/s11357-011-9246-0 PMC3312629

[32] Debras E, Prod'homme M, Rieu I, Balage M, Dardevet D, et al (2007) Postprandial leucine deficiency failed to alter muscle protein synthesis in growing and adult rats. Nutrition 23: 267–276 S0899-9007(06)00421-7 [pii];10.1016/j.nut.2006.12.003 [doi]. 17352963 10.1016/j.nut.2006.12.003

[33] Mosoni L, Patureau MP, Houlier ML, Arnal M (1993) Age-related changes in protein synthesis measured in vivo in rat liver and gastrocnemius muscle. Mech Ageing Dev 68: 209–220. 7688841 10.1016/0047-6374(93)90152-h

[34] Dardevet D, Sornet C, Balage M, Grizard J (2000) Stimulation of in vitro rat muscle protein synthesis by leucine decreases with age. J Nutr 130: 2630–2635. 11053498 10.1093/jn/130.11.2630

[35] Dardevet D, Remond D, Peyron MA, Papet I, Savary-Auzeloux I, et al (2013) MuscleWasting and Resistance of Muscle Anabolism: The “Anabolic Threshold Concept” for Adapted Nutritional Strategies during Sarcopenia. The ScientificWorld Journal 2012 269531 DOI: 10.1100/2012/269531 10.1100/2012/269531 PMC3541599 23326214

[36] Nair KS (1995) Muscle protein turnover: methodological issues and the effect of aging. J Gerontol A Biol Sci Med Sci 50 Spec No: 107–112. 7493201 10.1093/gerona/50a.special_issue.107

[37] Short KR, Vittone JL, Bigelow ML, Proctor DN, Coenen-Schimke JM, et al (2005) Changes in myosin heavy chain mRNA and protein expression in human skeletal muscle with age and endurance exercise training. J Appl Physiol 99: 95–102 00129.2005 [pii];10.1152/japplphysiol.00129.2005 [doi]. 15746299 10.1152/japplphysiol.00129.2005

[38] Yarasheski KE, Pak-Loduca J, Hasten DL, Obert KA, Brown MB, et al (1999) Resistance exercise training increases mixed muscle protein synthesis rate in frail women and men >/ = 76 yr old. Am J Physiol 277: E118–E125. 10409135 10.1152/ajpendo.1999.277.1.E118

[39] Rennie MJ, Wackerhage H, Spangenburg EE, Booth FW (2004) Control of the size of the human muscle mass. Annu Rev Physiol 66: 799–828 10.1146/annurev.physiol.66.052102.134444 [doi]. 14977422 10.1146/annurev.physiol.66.052102.134444

[40] Wolfe RR (2002) Regulation of muscle protein by amino acids. J Nutr 132: 3219S–3224S. 12368421 10.1093/jn/131.10.3219S

[41] Biolo G, Tipton KD, Klein S, Wolfe RR (1997) An abundant supply of amino acids enhances the metabolic effect of exercise on muscle protein. Am J Physiol 273: E122–E129. 9252488 10.1152/ajpendo.1997.273.1.E122

[42] Bohe J, Low JF, Wolfe RR, Rennie MJ (2001) Latency and duration of stimulation of human muscle protein synthesis during continuous infusion of amino acids. J Physiol 532: 575–579 PHY_12181 [pii]. 11306673 10.1111/j.1469-7793.2001.0575f.x PMC2278544

[43] Carroll CC, Fluckey JD, Williams RH, Sullivan DH, Trappe TA (2005) Human soleus and vastus lateralis muscle protein metabolism with an amino acid infusion. Am J Physiol Endocrinol Metab 288: E479–E485 00393.2004 [pii];10.1152/ajpendo.00393.2004 [doi]. 15507532 10.1152/ajpendo.00393.2004

[44] Garlick PJ (2005) The role of leucine in the regulation of protein metabolism. J Nutr 135: 1553S–1556S 135/6/1553S [pii]. 15930468 10.1093/jn/135.6.1553S

[45] May ME, Buse MG (1989) Effects of branched-chain amino acids on protein turnover. Diabetes Metab Rev 5: 227–245. 2656154 10.1002/dmr.5610050303

[46] Mittendorfer B, Andersen JL, Plomgaard P, Saltin B, Babraj JA, et al (2005) Protein synthesis rates in human muscles: neither anatomical location nor fibre-type composition are major determinants. J Physiol 563: 203–211 jphysiol.2004.077180 [pii];10.1113/jphysiol.2004.077180 [doi]. 15611031 10.1113/jphysiol.2004.077180 PMC1665563

[47] Verhoeven S, Vanschoonbeek K, Verdijk LB, Koopman R, Wodzig WK, et al (2009) Long-term leucine supplementation does not increase muscle mass or strength in healthy elderly men. Am J Clin Nutr 89: 1468–1475 ajcn.2008.26668 [pii];10.3945/ajcn.2008.26668 [doi]. 19321567 10.3945/ajcn.2008.26668

[48] Boirie Y, Dangin M, Gachon P, Vasson MP, Maubois JL, et al (1997) Slow and fast dietary proteins differently modulate postprandial protein accretion. Proc Natl Acad Sci U S A 94: 14930–14935. 9405716 10.1073/pnas.94.26.14930 PMC25140

[49] Dangin M, Boirie Y, Garcia-Rodenas C, Gachon P, Fauquant J, et al (2001) The digestion rate of protein is an independent regulating factor of postprandial protein retention. Am J Physiol Endocrinol Metab 280: E340–E348. 11158939 10.1152/ajpendo.2001.280.2.E340

[50] Dangin M, Boirie Y, Guillet C, Beaufrere B (2002) Influence of the protein digestion rate on protein turnover in young and elderly subjects. J Nutr 132: 3228S–3233S. 12368423 10.1093/jn/131.10.3228S

[51] Dardevet D, Remond D, Peyron MA, Papet I, Savary-Auzeloux I, et al (2012) Muscle Wasting and Resistance of Muscle Anabolism: The “Anabolic Threshold Concept” for Adapted Nutritional Strategies during Sarcopenia. The ScientificWorld Journal 2012: 269531. 23326214 10.1100/2012/269531 PMC3541599

[52] Moghaddam E, Vogt JA, Wolever TM (2006) The effects of fat and protein on glycemic responses in nondiabetic humans vary with waist circumference, fasting plasma insulin, and dietary fiber intake. J Nutr 136: 2506–2511 136/10/2506 [pii]. 16988118 10.1093/jn/136.10.2506

[53] Nilsson M, Stenberg M, Frid AH, Holst JJ, Bjorck IM (2004) Glycemia and insulinemia in healthy subjects after lactose-equivalent meals of milk and other food proteins: the role of plasma amino acids and incretins. Am J Clin Nutr 80: 1246–1253 80/5/1246 [pii]. 15531672 10.1093/ajcn/80.5.1246

[54] Petersen BL, Ward LS, Bastian ED, Jenkins AL, Campbell J, et al (2009) A whey protein supplement decreases post-prandial glycemia. Nutr J 8: 47 1475-2891-8-47 [pii];10.1186/1475-2891-8-47 [doi]. 19835582 10.1186/1475-2891-8-47 PMC2766379

